# Acute Mechanical Stretch Promotes eNOS Activation in Venous Endothelial Cells Mainly via PKA and Akt Pathways

**DOI:** 10.1371/journal.pone.0071359

**Published:** 2013-08-14

**Authors:** Zhenqian Hu, Yan Xiong, Xiaofan Han, Chenyang Geng, Beibei Jiang, Yingqing Huo, Jincai Luo

**Affiliations:** Laboratory of Vascular Biology, Institute of Molecular Medicine, Peking University, Beijing Key Laboratory of Cardiometabolic Molecular Medicine, Beijing, China; Ohio State University, United States of America

## Abstract

In the vasculature, physiological levels of nitric oxide (NO) protect against various stressors, including mechanical stretch. While endothelial NO production in response to various stimuli has been studied extensively, the precise mechanism underlying stretch-induced NO production in venous endothelial cells remains incompletely understood. Using a model of continuous cellular stretch, we found that stretch promoted phosphorylation of endothelial NO synthase (eNOS) at Ser^1177^, Ser^633^ and Ser^615^ and NO production in human umbilical vein endothelial cells. Although stretch activated the kinases AMPKα, PKA, Akt, and ERK1/2, stretch-induced eNOS activation was only inhibited by kinase-specific inhibitors of PKA and PI3K/Akt, but not of AMPKα and Erk1/2. Similar results were obtained with knockdown by shRNAs targeting the PKA and Akt genes. Furthermore, inhibition of PKA preferentially attenuated eNOS activation in the early phase, while inhibition of the PI3K/Akt pathway reduced eNOS activation in the late phase, suggesting that the PKA and PI3K/Akt pathways play distinct roles in a time-dependent manner. Finally, we investigated the role of these pathways in stretch-induced endothelial exocytosis and leukocyte adhesion. Interestingly, we found that inhibition of the PI3K/Akt pathway increased stretch-induced Weibel-Palade body exocytosis and leukocyte adhesion, while inhibition of the PKA pathway had the opposite effects, suggesting that the exocytosis-promoting effect of PKA overwhelms the inhibitory effect of PKA-mediated NO production. Taken together, the results suggest that PKA and Akt are important regulators of eNOS activation in venous endothelial cells under mechanical stretch, while playing different roles in the regulation of stretch-induced endothelial exocytosis and leukocyte adhesion.

## Introduction

The free radical nitric oxide (NO), produced by endothelial NO synthase (eNOS), is an important vasoactive substance in normal vascular biology and pathophysiology. In addition to its well-known vascular functions such as vessel dilation and angiogenesis [1], [2], NO also regulates some of the key steps in thrombosis and inflammation, including platelet aggregation and monocyte adhesion [3], [4]. In endothelial cells (ECs), NO production by eNOS is stimulated by a variety of chemical substances such as vascular endothelial growth factor, thrombin, hydrogen peroxide and bradykinin, as well as by hemodynamic forces, including shear stress, transmural pressure, and mechanical stretch [5]–[10].

While the molecular mechanisms underlying eNOS activation and NO production in arterial ECs in response to chemical stimuli and shear stress have been studied extensively, little is known about the mechanism in venous ECs under continuous stretch. Actually, continuous stretch of venous ECs caused by the abrupt and sustained dilation of veins is frequently observed in patients with portal vein embolization, venous congestion due to acute heart failure, and venous-arterial grafts [11]–[14]. In addition, over-stretch of venous ECs may be closely associated with venous thrombosis and inflammation [15]. Accumulated evidence suggests that mechanical stretch can induce an inflammatory response in endothelial cells [16], [17]. Endothelial exocytosis of Weibel-Palade bodies (WPBs), which contain von Willebrand factor (vWF), interleukin-8 (IL-8) and P-selectin, appears to be one of earliest events in the process of vascular inflammation [18], [19]. Recently, we showed that acute hypertensive stretch induces endothelial exocytosis and initiates the pro-thrombotic and pro-inflammatory responses of ECs [20]. On the other hand, NO production has inhibitory effects on venous thrombosis and inflammation [21], [22]. A previous study indicated that NO inhibits the endothelial exocytosis of WPBs *via* S-nitrosylation of N-Ethylmaleimide-sensitive Factor (NSF) [23]. Our recent study demonstrated that NO is also involved in the inhibition of stretch-induced endothelial exocytosis and vascular inflammation [20]. However, it is still unclear how stretch activates eNOS.

It is known that Ser^1177^ phosphorylation leads to increased eNOS activity and NO production [24]. So far, a series of protein kinases, including PKB/Akt, protein kinase A (PKA), PKG, AMP-activated protein kinase (AMPK), mitogen-activated protein kinase (MAPK) and calmodulin-dependent kinase II, has been shown to regulate the Ser^1177^ phosphorylation of eNOS [25]–[30]. In addition to Ser^1177^, eNOS has several other potential phosphorylation sites, including Ser^615^ and Ser^633^, the phosphorylation of which enhances the activity of eNOS. It has been shown that Ser^615^ is phosphorylated in a PKB/Akt-dependent manner while Ser^633^ in a PKA-dependent manner [31], [32]. These results provide clues for investigating the regulatory pathways of stretch-induced eNOS activation and NO production in venous ECs.

Therefore, we set out to determine whether AMPK, Akt, PKA, and MAPK regulate the Ser^1177^ phosphorylation of eNOS and NO production in human umbilical vein endothelial cells (HUVECs) under continuous stretch by using kinase-specific inhibitors and gene-specific shRNAs.

## Results

### Stretch Stimulates eNOS Activation and NO Production in Venous ECs

**Figure 1:**
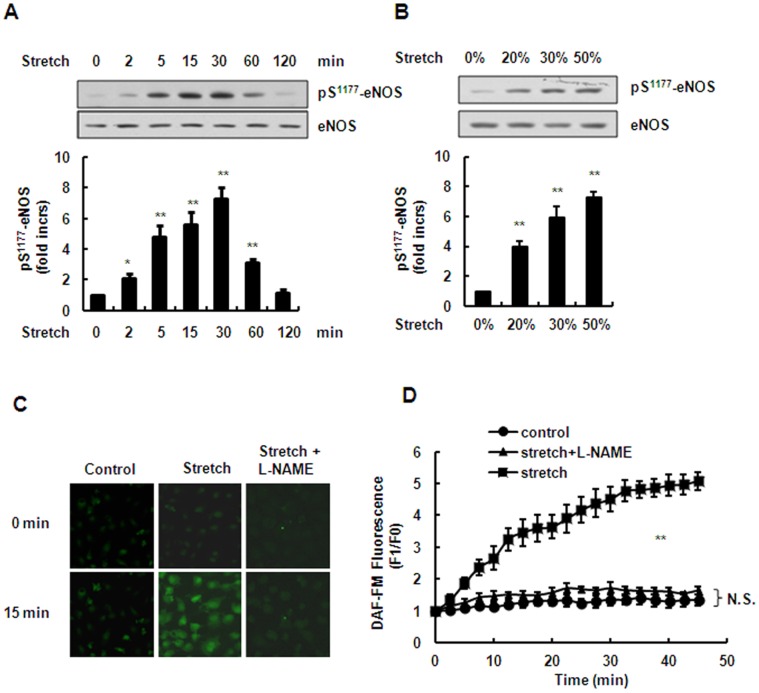
Effects of stretch on eNOS activation and NO production. (A) HUVECs were subjected to 50% stretch for the times indicated (0, 2, 5, 15, 30, 60 and 120 min). Phosphorylation of eNOS in cell lysates was analyzed by immunoblotting with phospho-eNOS (Ser^1177^). The same blot was stripped and re-probed with antibody detecting total eNOS to monitor the equal loading of samples (*upper*), and the quantitative analysis of Ser^1177^ phosphorylation for eNOS was normalized by arbitrarily setting the density of control cells (time = 0) to 1.0 (*lower*). (B) *Upper*: Western blots of phospho-eNOS (Ser^1177^) in HUVECs stretched to the indicated magnitudes for 15 min. *Lower*: quantitative analysis of Ser^1177^ phosphorylation of eNOS. (C) DAF-FM staining was performed to detect NO release under continuous stretch (time = 15 min) and 1 mM L-NAME (a NOS inhibitor) was used to pre-treat HUVECs for 1 h. (D) Quantitative analysis of NO release under continuous stretch. Results are representative of 3 individual experiments and expressed as mean ± S.D. (n = 4). *p<0.05; **p<0.01; N.S., not significant.

We first confirmed the effect of stretch on the Ser^1177^ phosphorylation of eNOS and NO production. Early reports suggest that under shear stress, sustained eNOS activation for as long as 30–60 min was detected [26], [36]. Therefore, we examined eNOS activation in ECs under stretch for as long as 120 min. The result showed that stretch induced Ser^1177^ phosphorylation of eNOS in a time-dependent manner without significant change in the amount of total eNOS protein and had no significant effect on cell death within 2 h (Fig. 1A and Fig. S1). The Ser^1177^ phosphorylation was apparent as early as 2 min after stretch and reached a peak at 30 min, detectable at 60 min and returned to the base level at 120 min. HUVECs were then subjected to mechanical stretch for 15 min for different magnitudes (20%–50%). We found that stretch induced the Ser^1177^ phosphorylation in a magnitude-dependent manner (Figure 1B). As the phosphorylation of eNOS Ser^1177^ is critical for NO production, we used DAF-FM (an NO species indicator) to assess the NO levels in HUVECs. The NO levels were significantly increased after stretch compared with the control. In addition, L-NAME (a NOS inhibitor) significantly inhibited the stretch-induced NO production (Fig. 1C, 1D).

### Stretch Stimulates Phosphorylation of AMPKα, Akt, Erk1/2 and the Activation of PKA

**Figure 2:**
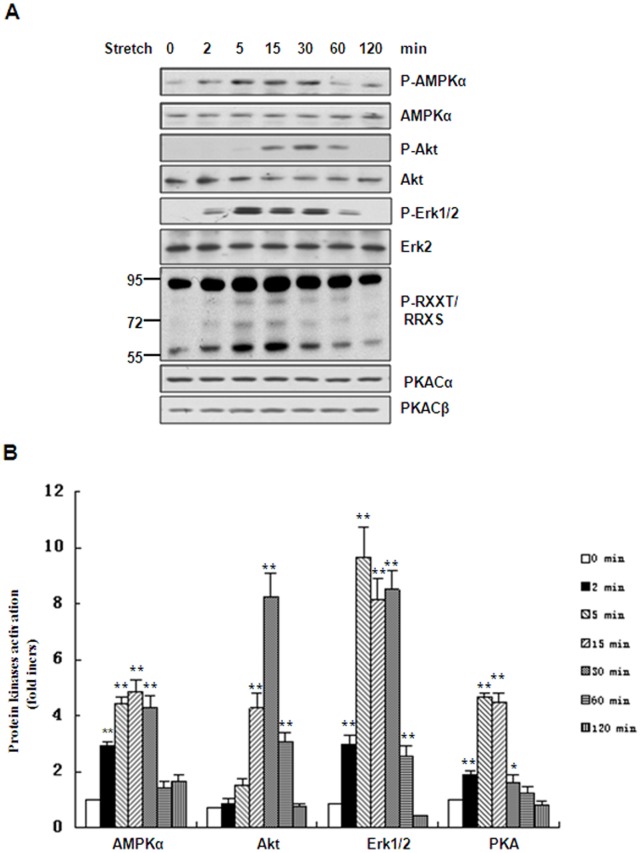
Effects of stretch on phosphorylation of AMPKα, Akt, Erk1/2 and activation of PKA. **(**A) Western blots of phospho-AMPKα (Thr^172^), phospho-Akt (Ser^473^), phospho-Erk1/2 (Thr^202^/Tyr^204^) and PKA substrates in HUVECs stretched for the indicated times. The same blot was stripped and re-probed with antibodies detecting the total amount of each protein to monitor equal loading of samples. (B) Quantitative analysis of stretch-induced phosphorylation or activation of protein kinases for the times indicated. Results are representative of 3 individual experiments and expressed as mean ± S.D. (n = 4). *p<0.05; **p<0.01.

Sustained eNOS activation by stretch as mentioned above prompted us to identify the kinases responsible for eNOS activation and their nature. Previous work has shown that AMPKα, Akt, Erk1/2 and PKA phosphorylate eNOS [24], [25], [29], [33]. Therefore, we determined whether these kinases are activated by mechanical stretch. Under static conditions, the phosphorylation of AMPK on Thr^172^, Akt on Ser^473^and Erk1/2 on Thr^202^/Tyr^204^ was relatively low. The phosphorylation of these kinases was evident after mechanical stretch but showed different time courses. The phosphorylation of AMPK and Erk1/2 increased as early as 2 min and peaked from 5 to 30 min, while that of Akt was delayed to 15 min, peaked at 30 min, and then returned to the control level at 2 h. In addition, we measured PKA activity using a phospho-PKA substrate antibody, and found that it peaked at 5 min and 15 min after mechanical stretch, returning to the control level at 1 h (Figure 2A, B).

### AMPKα and ERK Pathways are not Involved in the Regulation of Stretch-induced Ser^1177^ Phosphorylation of eNOS and NO Production

**Figure 3:**
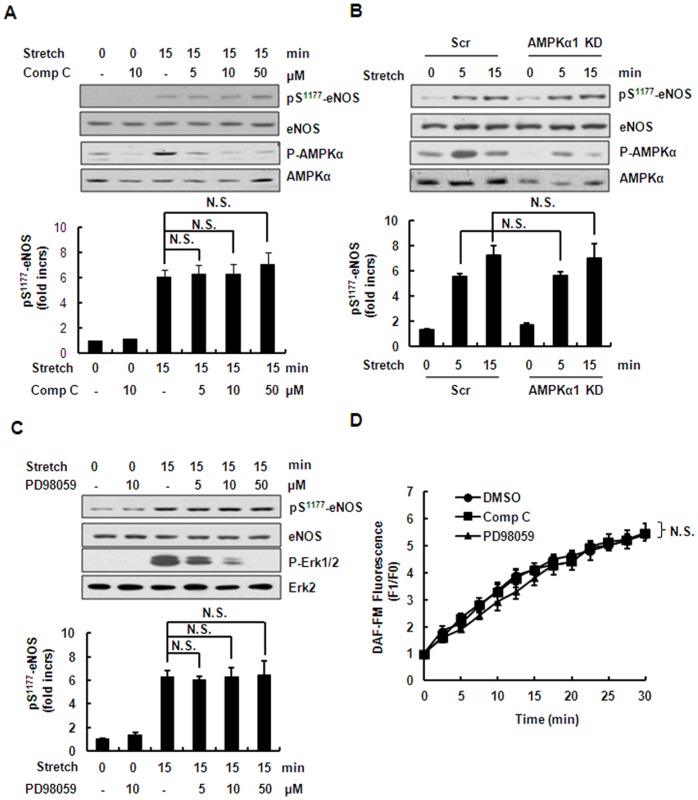
AMPKα and ERK pathways are not involved in stretch-induced phosphorylation of eNOS. **(**A) *Upper*: Western blots of phospho-eNOS (Ser^1177^) and phospho-AMPKα (Thr^172^) in HUVECs under continuous stretch, pretreated with Compound C (5–50 µM). *Lower*: Quantitative analysis of Ser^1177^ phosphorylation of eNOS. (B) *Upper*: Western blots of phospho-AMPKα (Thr^172^) and phospho-eNOS (Ser^1177^) in HUVECs expressing scrambled (Scr) or AMPKα1-targeting (AMPKα1 KD) shRNAs under continuous stretch. *Lower*: Quantitative analysis of Ser^1177^ phosphorylation of eNOS. (C) *Upper*: Western blots of phospho-Erk1/2 (Thr^202^/Tyr^204^) and phospho-eNOS (Ser^1177^) in HUVECs after stretch, pretreated with PD98059 (5–50 µM). *Lower*: Quantitative analysis of Ser^1177^ phosphorylation of eNOS. (D) Quantitative analysis of NO release in HUVECspretreated with 10 µM Compound C or PD980598, under continuous stretch. Results are representative of 3 individual experiments and expressed as mean ± S.D. (n = 4). N.S., not significant.

The above results showing stretch-induced phosphorylation or activation of protein kinases prompted us to consider whether these kinases regulate the Ser^1177^ phosphorylation of eNOS and NO production. We thus chose to use kinase-specific inhibitors and gene-specific shRNAs to investigate their regulation of eNOS phosphorylation. First, HUVECs were pretreated for 30 min with 5–50 µM Compound C, a highly-selective inhibitor of AMPKα, and then stretched for 15 min. Compound C had no significant effect on stretch-induced Ser^1177^ phosphorylation of eNOS, while it inhibited stretch-induced phosphorylation of AMPKα in a dose-dependent manner (Fig. 3A). Then we used specific shRNA targeting the AMPKα1 gene and found that knock-down of AMPKα1 had a similar effect on Ser^1177^ phosphorylation of eNOS (Fig. 3B). In addition, PD98059, a selective inhibitor of MEK1/2, inhibited stretch-induced phosphorylation of Erk1/2 in a dose-dependent manner but had no significant effect on the Ser^1177^ phosphorylation of eNOS (Fig. 3C). Furthermore, the inhibition of AMPKα and Erk1/2 had no significant effect on stretch-induced NO production (Fig. 3D). These results suggested that the AMPKα and ERK pathways are dispensable for regulation of stretch-induced Ser^1177^ phosphorylation of eNOS.

### PKA Pathway Mediates Stretch-induced Ser^1177^ Phosphorylation of eNOS and NO Production in the Early Phase

**Figure 4:**
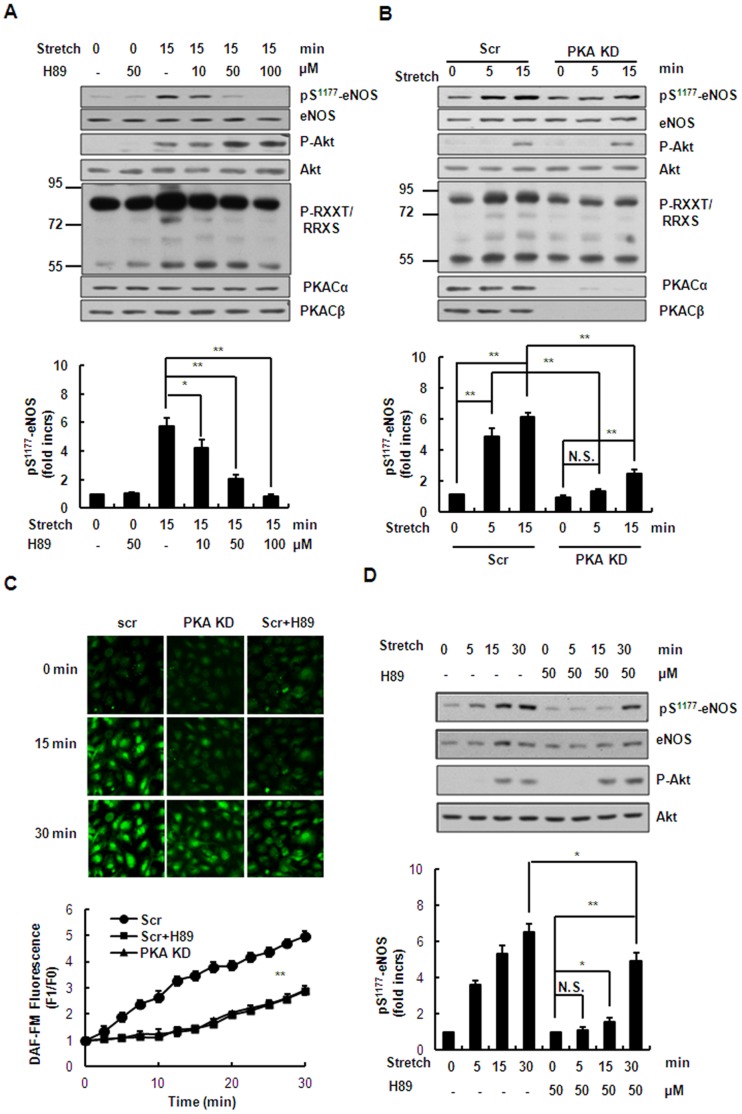
PKA pathway mediates stretch-induced phosphorylation of eNOS and NO production in the early phase. **(**A) *Upper*: Western blots of phospho-Akt (Ser^473^), phospho-eNOS (Ser^1177^) and PKA substrates in HUVECs under continuous stretch (15 min), pretreated with H89 (10–100 µM). *Lower*: quantitative analysis of Ser^1177^ phosphorylation of eNOS. (B) *Upper*: Western blots of phospho-Akt (Ser^473^), phospho-eNOS (Ser^1177^) and PKA substrates in HUVECs expressing scrambled (Scr) or PKA-targeting (PKA KD) shRNAs under continuous stretch. *Lower*: quantitative analysis of Ser^1177^ phosphorylation of eNOS. (C) *Upper*: DAF-FM staining of HUVECs expressing scrambled (Scr) or PKA-targeting (PKA KD) shRNAs with or without 50 µM H89 pretreatment, under continuous stretch. *Lower*: quantitative analysis of NO release. (D) *Upper*: Western blots of phospho-Akt (Ser^473^) and phospho-eNOS (Ser^1177^) in HUVECs pretreated with or without 50 µM H89 under continuous stretch for the indicated time periods. *Lower*: quantitative analysis of Ser^1177^ phosphorylation of eNOS. Results are representative of 3 individual experiments and expressed as mean ± S.D. (n = 4). *p<0.05; **p<0.01.

To determine whether stretch-induced Ser^1177^ phosphorylation of eNOS and NO production is regulated by a PKA-dependent mechanism, HUVECs were pre-treated for 1 h with 10–100 µM H89, a PKA-specific inhibitor, and then stretched for 15 min. Treatment of the cells with H89 significantly attenuated the Ser^1177^ phosphorylation of eNOS in a dose-dependent manner, while slightly increasing the phosphorylation of Akt (Fig. 4A). Furthermore, specific shRNAs targeting both PKA catalytic subunits α and β greatly reduced the stretch-induced Ser^1177^ phosphorylation of eNOS and slightly increased the phosphorylation of Akt compared with the scrambled control (Fig. 4B), confirming a role of PKA in regulating eNOS activation and the interplay between PKA and Akt. In accord with these results, NO production was significantly inhibited in HUVECs pretreated with 50 µM H89 or expressing shRNAs targeting both PKA catalytic subunits after stretch for 15 min, compared with scrambled control (Fig. 4C). These results suggested that stretch induces Ser^1177^ phosphorylation of eNOS and NO production in the early phase (≤15 min) in a PKA-dependent manner. Interestingly, after stretch for >15 min Ser^1177^ phosphorylation of eNOS and NO production were not completely abolished but still slightly increased in the presence of 50 µM H89 or expressing the shRNAs targeting both PKA subunits, compared with unstretched cells (Fig. 4C–D). These results suggested that stretch induces Ser^1177^ phosphorylation of eNOS and NO production in the late phase in a PKA-independent manner.

### PI3K/Akt Pathway Mediates Stretch-induced Ser^1177^ Phosphorylation of eNOS and NO Production in the Late Phase

**Figure 5:**
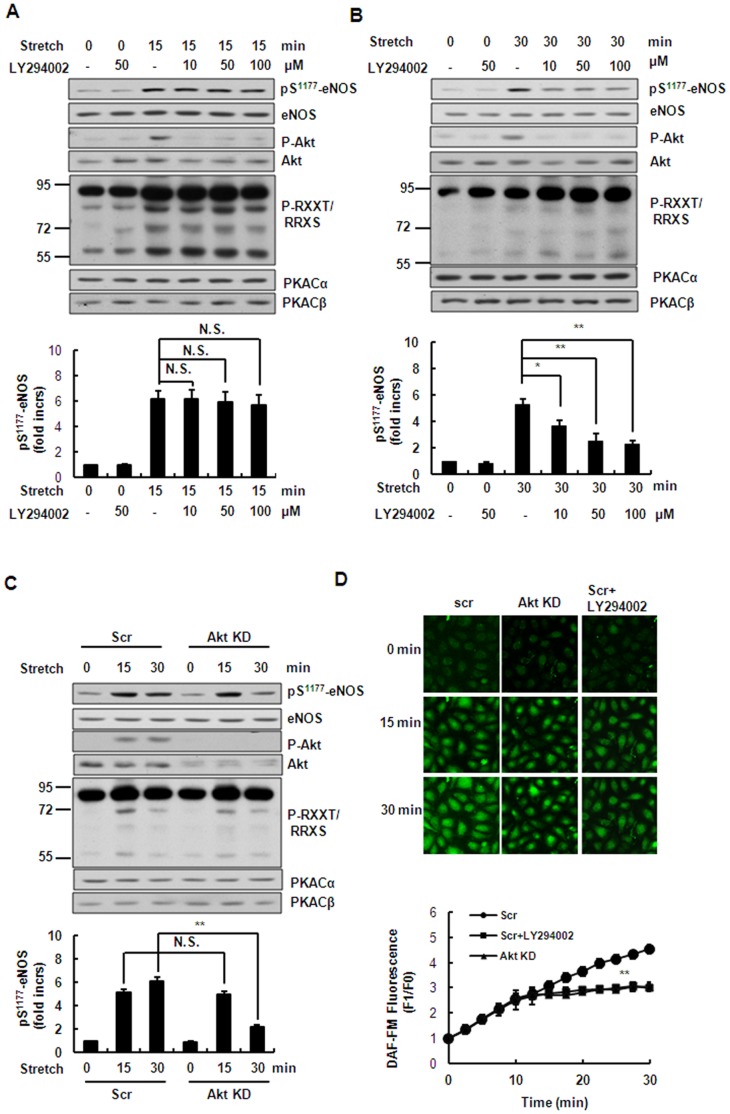
PI3K/Akt pathway mediates stretch-induced phosphorylation of eNOS and NO production in the late phase. **(**A) *Upper*: Western blots of phospho-Akt (Ser^473^), phospho-eNOS (Ser^1177^), and phospho-PKA substrates in HUVECs under continuous stretch (15 min), pretreated with LY294002 (10–100 µM). *Lower*: quantitative analysis of Ser^1177^ phosphorylation of eNOS. (B) Western blots of phospho-Akt (Ser^473^), phospho-eNOS (Ser^1177^), and PKA substrates in HUVECs under continuous stretch (30 min), pretreated with LY294002 (10–100 µM). *Lower*: quantitative analysis of Ser^1177^ phosphorylation of eNOS. (C) *Upper*: Western blots of phospho-Akt (Ser^473^), phospho-eNOS (Ser^1177^), and PKA substrates in HUVECs expressing scrambled (Scr) or Akt1/2-targeting (Akt KD) shRNAs under continuous stretch. *Lower*: quantitative analysis of Ser^1177^ phosphorylation of eNOS. (D) *Upper*: DAF-FM staining of HUVECs expressing scrambled (Scr) or Akt1/2-targeting (Akt KD) shRNAs with or without 50 µM LY294002 pretreatment, under continuous stretch *Lower*: quantitative analysis of NO release. Results are representative of 3 individual experiments and expressed as mean ± S.D. (n = 4). *p<0.05; **p<0.01; N.S., not significant.

The above results showed that the stretch-induced phosphorylation of Akt was relatively delayed. Therefore we speculated that Akt would regulate the late phase of stretch-induced Ser^1177^ phosphorylation of eNOS and NO production. First, HUVECs were pretreated for 30 min with 10–100 µM LY294002 (a PI3K inhibitor), and then subjected to stretch for 15 min. The LY294002 treatment abolished the stretch-induced phosphorylation of Akt in a dose-dependent manner but had no significant effect on the phosphorylation of eNOS under these conditions (Fig. 5A). However, at 30 min of stretch, LY294002 attenuated the stretch-induced Ser^1177^ phosphorylation of eNOS in a dose-dependent manner (Fig. 5B). Then, specific shRNAs targeting both Akt1 and Akt2 significantly reduced the Ser^1177^ phosphorylation of eNOS at 30 min of stretch but had no significant effect at 15 min of stretch compared with scrambled control (Fig. 5C). Furthermore, NO production did not change after stretching for 15 min but decreased after stretching for 30 min in HUVECs pretreated with 50 µM LY294002 or expressing the shRNAs targeting Akt1 and Akt2, compared with scrambled control (Fig. 5D). The above results suggested that stretch induces the late phase of eNOS Ser^1177^ phosphorylation and NO production in an Akt-dependent manner.

### Stretch Induces Ser^633^ Phosphorylation of eNOS in a PKA-dependent Manner and Ser^615^ Phosphorylation in a PI3K/Akt-dependent Manner

**Figure 6:**
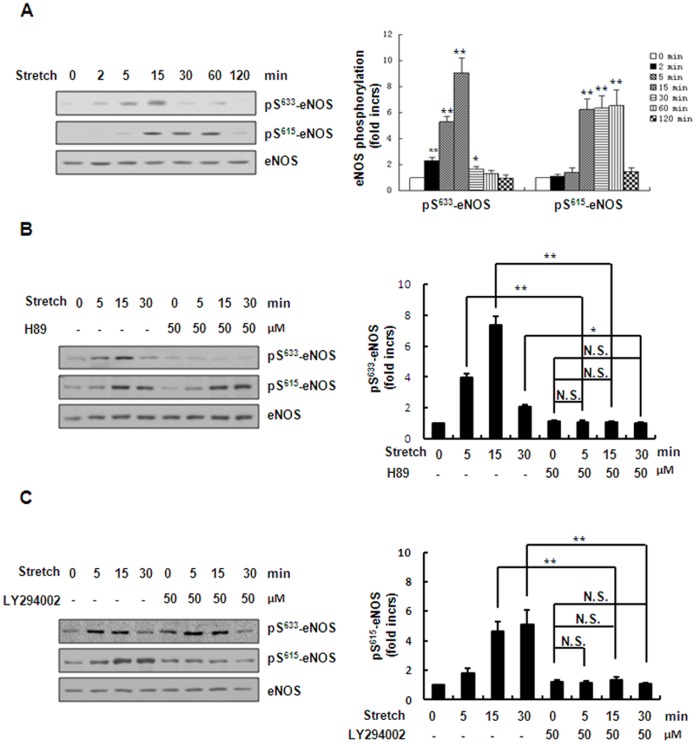
PKA and PI3K/Akt pathways respectively mediate stretch-induced eNOS-Ser^633^ and Ser^615^ phosphorylation. (A) *Left*: Western blots of Ser^633^ and Ser^615^ phosphorylation of eNOS in HUVECs under continuous stretch (50%) for the times indicated. *Right*: quantitative analysis of Ser^633^and Ser^615^phosphorylation of eNOS. (B) *Left*: Western blots of Ser^633^ and Ser^615^ phosphorylation of eNOS in HUVECs under continuous stretch (50%) for the times indicated, pretreated with or without 50 µM H89. *Right*: quantitative analysis of Ser^633^ and Ser^615^ phosphorylation of eNOS. (C) *Left*: Western blots of Ser^633^ and Ser^615^ phosphorylation of eNOS in HUVECs under continuous stretch (50%) for the times indicated, pretreated with or without 50 µMLY294002. *Right*: quantitative analysis of Ser^633^ and Ser^615^ phosphorylation of eNOS. Results are representative of 3 individual experiments and expressed as mean ± S.D. (n = 4). *p<0.05; **p<0.01; N.S., not significant.

Previous work has shown that there are other potential phosphorylation sites in eNOS, including Ser^633^ and Ser^615^ [31], [32]. Therefore, we determined whether these two sites are phosphorylated by mechanical stretch. Under continuous stretch, the Ser^633^ phosphorylation increased as early as 2 min and peaked at 15 min, while the Ser^615^ phosphorylation was relatively delayed, peaked from 15 min to 60 min (Fig. 6A). Then, we used the above kinase-specific inhibitors to determine the role of these kinases in regulation of stretch-induced Ser^633^ and Ser^615^ phosphorylation of eNOS. We found that inhibition of the PKA pathway using H89 abolished the stretch-induced Ser^633^ phosphorylation but did not attenuate the stretch-induced Ser^615^ phosphorylation (Fig. 6B). Furthermore, inhibition of the PI3K/Akt pathway using LY294002 abolished the stretch-induced Ser^615^ phosphorylation but did not affect the stretch-induced Ser^633^ phosphorylation (Fig. 6C). The above results demonstrated that the PKA pathway mediates stretch-induced Ser^633^ phosphorylation of eNOS while the PI3K/Akt pathway mediates stretch-induced Ser^615^ phosphorylation.

### eNOS Activation and NO Production Negatively Regulate Stretch-induced WPB Exocytosis and Leukocyte Adhesion

**Figure 7:**
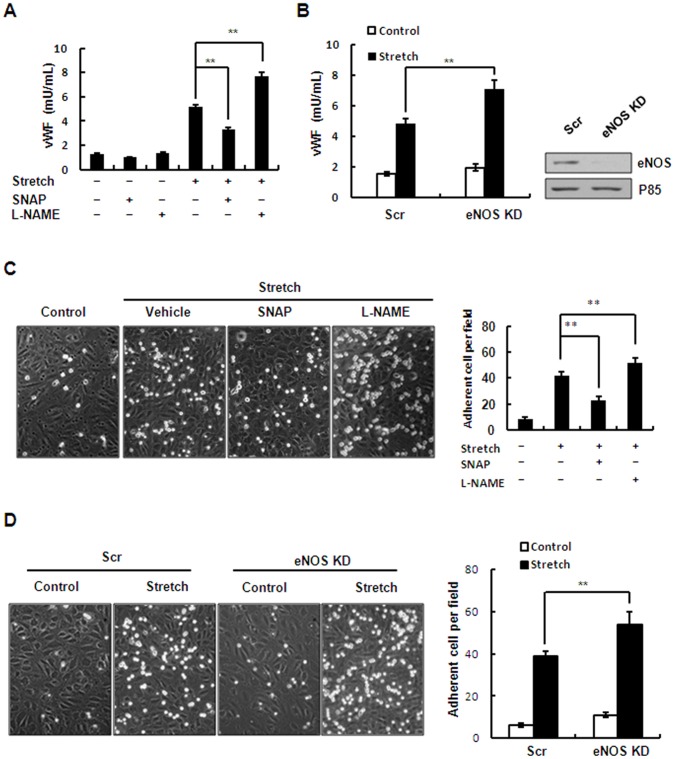
Effect of stretch-induced eNOS activation and NO production on WPB exocytosis and leukocyte adhesion. (A) Quantitative analysis of stretch-induced vWF release from HUVECs pretreated with SNAP (50 µM), L-NAME (1 mM) or vehicle (DMSO). (B) *Left*: quantitative analysis of stretch-induced vWF release from HUVECs expressing scrambled (Scr) or eNOS-targeting (eNOS KD) shRNAs. *Right*: Western blots of the knockdown efficiency of shRNAs targeting eNOS in HUVECs. (C) *Left*: HL-60 cell adhesion to HUVEC monolayers after stretch, pretreated with SNAP (50 µM), L-NANE (1 mM) or vehicle (DMSO). *Right*: quantitative analysis of HL-60 adhesion. (D) *Left*: stretch-induced HL-60 cell adhesion to HUVECs expressing scrambled (Scr) or eNOS-targeting (eNOS KD) shRNAs. *Right*: quantitative analysis of HL-60 adhesion. Results are representative of 3 individual experiments and expressed as mean ± S.D. (n = 4). **P<0.01.

NO has been shown to inhibit endothelial WPBs exocytosis, an early event in leukocyte adhesion [23]. We thus examined the effect of eNOS activation and NO production on stretch-induced endothelial WPB exocytosis and leukocyte adhesion by using chemical inhibitors and gene-specific RNA knockdown (Fig. 7A and 7C). Stretch significantly enhanced endothelial WPB exocytosis and leukocyte adhesion, consistent with our recent finding [20]. The increase in NO production by pretreatment of HUVECs with SNAP, an NO donor that provides exogenous NO, significantly attenuated the stretch-induced exocytosis and leukocyte adhesion. In contrast, the decrease in NO production by pretreatment of HUVECs with L-NAME, an inhibitor of NO production, intensified both of these processes (Fig. 7A, C). Consistently, the levels of stretch-induced exocytosis and adhesion were significantly intensified in HUVECs expressing the shRNAs of eNOS compared with scrambled control (Fig. 7B, D). Taken together, these results suggested that eNOS activation and NO production exert negative feedback on stretch-induced WPB exocytosis and leukocyte adhesion.

### Inhibition of the PI3K/Akt Pathway Increases Stretch-induced WPB Exocytosis and Leukocyte Adhesion While Inhibiting the cAMP/PKA Pathway has Opposite Effects

**Figure 8:**
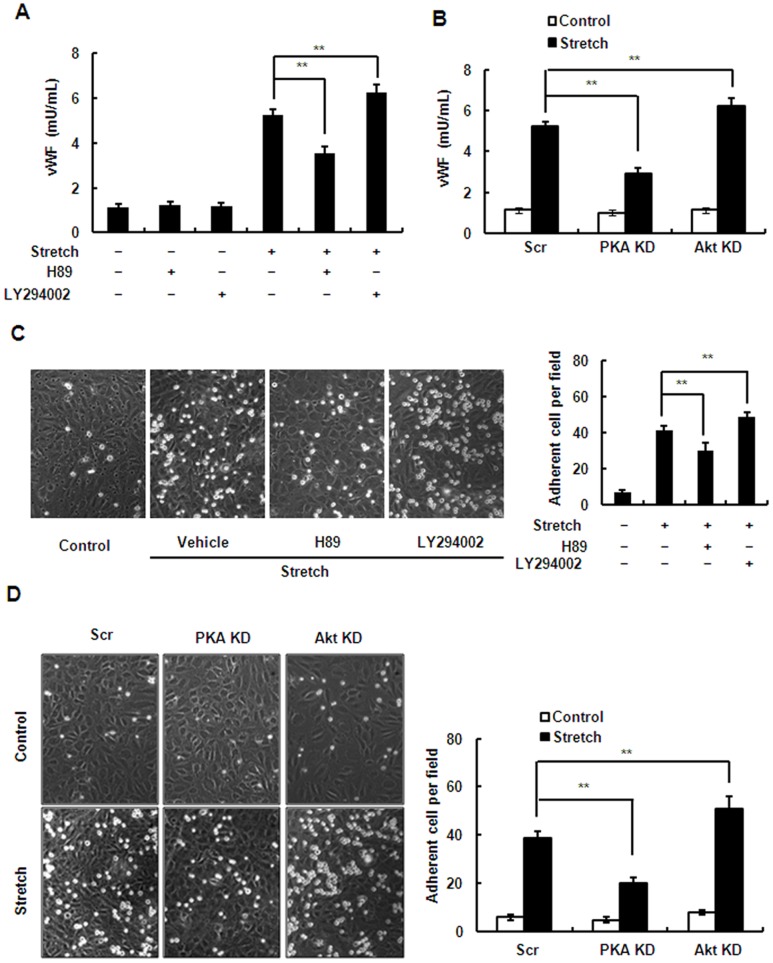
Inhibition of the PI3K/Akt pathway increases stretch-induced WPB exocytosis and leukocyte adhesion while inhibition of PKA has the opposite effect. (A) vWF release from HUVECs pretreated with H89 (50 µM), LY294002 (50 µM) or vehicle (DMSO) in response to stretch. (B) vWF release from HUVECs expressing scrambled (Scr), PKA-targeting (PKA KD), or Akt1/2-targeting (Akt KD) shRNA under continuous stretch. (C) *Left*: HL-60 cell adhesion to HUVEC monolayers after stretch, pretreated with H89 (50 µM), LY294002 (50 µM) or vehicle (DMSO). *Right*: quantitative analysis of HL-60 adhesion. (D) *Left*: HL-60 cell adhesion to HUVECs expressing scrambled (Scr), PKA-targeting (PKA KD), or Akt1/2-targeting (Akt KD) shRNA after stretch. *Right*: quantitative analysis of HL-60 adhesion. Results are representative of 3 individual experiments and expressed as mean ± S.D. (n = 4), **p<0.01.

**Figure 9:**
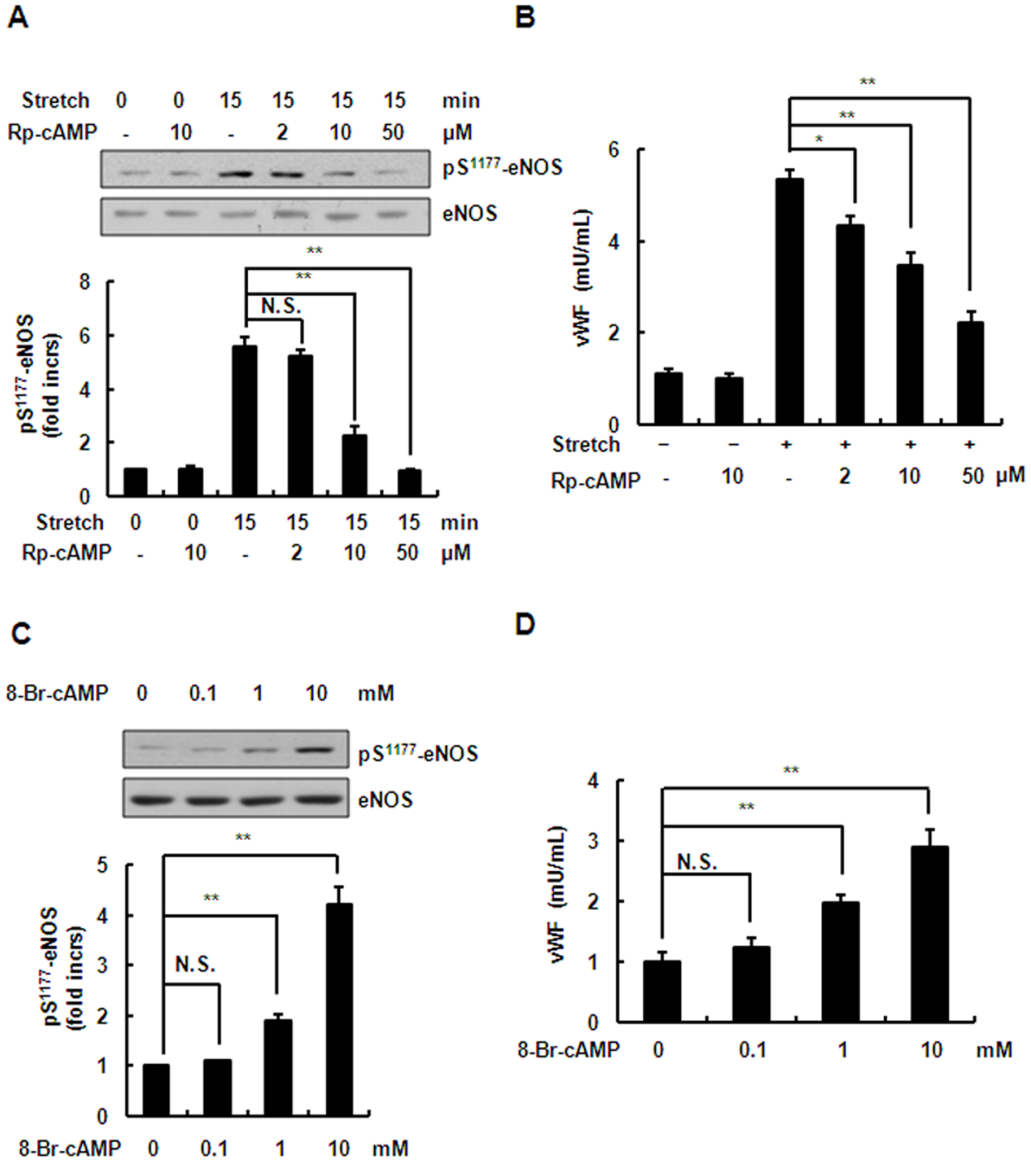
Effects of antagonist and agonist on phosphorylation of eNOS and vWF release. (A) *Upper*: Western blots of phospho-eNOS (Ser^1177^) in HUVECs under continuous stretch (15 min), pretreated with 5–50 µM Rp-cAMP (cAMP inhibitor). *Lower*: quantitative analysis of Ser^1177^ phosphorylation of eNOS. (B) Stretch-induced vWF release from HUVECs pretreated with 5–50 µM Rp-cAMP as indicated for 1 h. (C) *Upper*: Western blots of phospho-eNOS (Ser^1177^) in HUVECs in response to 0.1–10 mM 8-Br-cAMP for 5 min. *Lower*: quantitative analysis of Ser^1177^ phosphorylation of eNOS. (D) vWF release from HUVECs in response to 0.1–10 mM 8-Br-cAMP. Results are representative of 3 individual experiments and expressed as mean ± SD (n = 4). *P<0.05, **P<0.01; N.S., not significant.

Using chemical inhibitors and gene-specific RNA knockdown, we further studied the role of PKA- and PI3K/Akt-mediated eNOS activation and NO production in stretch-induced WBP exocytosis and leukocyte adhesion in endothelial cells. As expected, inactivation of Akt by both LY294002, an inhibitor of the PI3K/Akt pathway, and the shRNAs targeting Akt1 and Akt2, significantly intensified the stretch-induced exocytosis (Fig. 8A, B) and adhesion (Fig. 8C, D). Unexpectedly, the inhibition of PKA by both H89, a PKA-specific inhibitor, and the shRNAs targeting the catalytic subunits (α and β) of PKA significantly attenuated the stretch-induced exocytosis and adhesion (Fig. 8). The cAMP/PKA pathway has been shown to mediate WPB exocytosis [34]. We thus speculated that the exocytosis-promoting effect of cAMP/PKA overwhelms the inhibitory effect of PKA-mediated NO production. To confirm this, we further examined the roles of the cAMP/PKA pathway in eNOS activation and in WPB exocytosis of ECs under stretch. First, HUVECs were pretreated with 5–50 µM Rp-cAMP (a cAMP competitor) for 1 h, and then stretched. We found that Rp-cAMP inhibited the stretch-induced exocytosis even though the Ser^1177^ phosphorylation of eNOS was attenuated (Fig. 9A, B). Consistently, 0.1–10 mM 8-Br-AMP, an agonist for raising cAMP, significantly increased exocytosis as well as the Ser^1177^ phosphorylation of eNOS (Fig. 9C, D). These results indicated thatthe exocytosis-promoting effect of cAMP/PKAis stronger than the exocytosis-inhibiting effect of PKA-mediated NO production in ECs under stretch.

## Discussion

The most important finding in the current study was that in venous ECs, mechanical stretch induced Ser^1177^ phosphorylation of eNOS and NO production *via* the PKA and PI3K/Akt pathways in a time-dependent manner. The PKA pathway regulates Ser^1177^ phosphorylation of eNOS and NO production in the early phase and the PI3K/Akt pathway in the late phase. In addition, the PKA pathway mediates stretch-induced Ser^633^ phosphorylation of eNOS, while the PI3K/Akt pathway mediates stretch-induced Ser^615^ phosphorylation in ECs. Although stretch-induced NO production acts as a negative feedback on stretch-induced WBP exocytosis and leukocyte adhesion, the PKA pathway shows overwhelming positive regulation while the PI3K/Akt pathway still shows negative regulation.

Our conclusion that PKA and Akt kinases play distinctive roles in the activation of eNOS in a time-dependent manner is based on the following lines of evidence: (1) stretch-induced activation of PKA pathway occurred relatively earlier than Akt pathway (Fig. 2); (2) inhibition of PKA pathway by kinase-specific inhibitor as well as gene-specific shRNAs significantly attenuated Ser^1177^ phosphorylation of eNOS by stretch in the early phase (≤15 min), whereas inhibition of Akt pathway by the inhibitor as well as gene-specific shRNAs decreased Ser^1177^ phosphorylation of eNOS in the late phase (>15 min) (Fig. 4, 5 ); (3) PKA-mediated Ser^633^ phosphorylation of eNOS by stretch occurred relatively earlier (from 2 to 15 min), compared to Akt-mediated Ser^615^ phosphorylation of eNOS (from 15 to 60 min) (Fig. 6). Taken together, stretch-induced activation of eNOS in ECs is controlled through such time-dependent coordinated regulation of PKA and Akt.

One possible explanation for the biphasic response is that the difference of the two signaling pathways is caused different rates of activation by upstream pathways. Previous work has shown that VEGFR2 and GPCRs are components of the mechanosensor complex in ECs [35], [36]. We showed that a cAMP antagonist attenuates stretch-induced eNOS activation, and it is conceivable that the cAMP level might increase under continuous stretch *via* GPCRs. Some reports have shown that VEGFR2-mediated activation of the PI3K/Akt pathway by mechanical stress is relatively delayed (within minutes) [37], [38], compared with the GPCR-mediated activation of the PKA pathway that occurs relatively quickly (within seconds) [39], [40]. Another possible explanation is that PKA may have higher or prior affinity for eNOS than Akt for Ser^1177^ phosphorylation of eNOS. A slight increase of Ser^1177^ phosphorylation of eNOS can be detected under stretch for 15 min in HUVECs after inhibition of the PKA pathway, while inhibition of the PI3K/Akt pathway does not attenuate stretch-induced Ser^1177^ phosphorylation of eNOS at this time point (Figs. 4 and 5). This result suggested that the activation of PKA plays dominant role in phosphorylating eNOS-Ser^1177^ in the early phase (≤15 min) and when PKA is inhibited or deactivated (>15 min), Akt replaces PKA and plays the main role in maintaining Ser^1177^ phosphorylation of eNOS until the recruitment of Hsp90 in the eNOS complex [26], [41]. It should be noted that stretch-induced eNOS activation and NO production by the PKA and Akt pathways were not absolutely separate. In fact, there was still some overlap between activation of the two pathways (Fig. 2).

In addition, our work showed that acute stretch induced Ser^1177^ phosphorylation in an AMPK-independent manner, although phosphorylation of AMPK was also significantly increased (Figs. 2 and 3). Accumulating evidence demonstrates that AMPK directly phosphorylates eNOS Ser^1177^ [42]. It has been reported that AMPK is also involved in shear stress-dependent eNOS activation [33]. The discrepancy might be due to the different features of shear stress and stretch. Previous work has demonstrated that shear stress is a protective stimulus [43], but over-stretch of ECs injures blood vessels. Thus, there might be different signaling pathways at play, due to the availability and kinetics of competing eNOS-activating kinases, and their reaction rates. In addition, it has also been suggested that AMPK activation alone might not be sufficient to trigger Ser^1177^phosphorylation of eNOS under certain conditions. For example, Thors *et al* found that eNOS becomes AMPK-responsive under conditions of ATP depletion but not when cellular ATP is high [44]. Thus, care should be taken when interpreting the role of AMPK in eNOS activation.

The present study suggested that stretch-induced eNOS activation and NO production attenuated but did not abolish stretch-induced WPB exocytosis and leukocyte adhesion and acted as an auto-negative feedback for stretch-induced vascular inflammation. Thus we explored the role of cAMP/PKA- and PI3K/Akt-mediated NO production in stretch-induced WPB exocytosis and leukocyte adhesion. Interestingly, inhibition of the PI3K/Akt pathway increased the stretch-induced exocytosis and adhesion, while inhibition of the PKA pathway unexpectedly had the opposite effect (Fig. 8). Previous work has shown that the cAMP/PKA pathway also positively regulates WPB exocytosis *via* RalGDS [34]. It is most likely that the exocytosis-promoting effect of cAMP/PKA overwhelms the exocytosis-inhibiting effect of PKA-mediated NO production. Thus, the effect of PKA-mediated NO production is not evident in the regulation of stretch-induced WPB exocytosis and leukocyte adhesion. However, a negative role of PKA-mediated NO production is not excluded. In addition, NO has other physiological actions, such as short-term vessel dilation and long-term apoptosis, which need further investigation.

In conclusion, the current study demonstrated that time-dependent coordinated regulation of PKA and Akt kinase pathways is critical for the regulation of eNOS activation and NO production. Our results may provide a novel insight into the protective mechanism against vascular inflammation by mechanical stretch under pathological conditions in the early stage.

## Materials and Methods

### Reagents

Rabbit polyclonal antibodies to Akt, PKACα, PKACβ and Erk2 were from Santa Cruz Biotechnology (Santa Cruz, CA). Mouse monoclonal antibodies to phospho-eNOS (Ser^1177^) and phospho-eNOS (Ser^633^) (for detecting eNOS activation) and eNOS were from BD Biosciences (San Diego, CA). Rabbit antibody to phospho-eNOS (Ser^615^) was from Upstate (Millipore). Rabbit polyclonal antibodies to phospho-PKA substrate, phospho-Akt (Ser^473^) (for detecting Akt activation), phospho-AMPKα (Thr^172^) (for detecting AMPKα activation), AMPKα and phosphor-MEK1/2 (Thr^202^/Tyr^204^) were from Cell Signaling Technology (Beverly, MA). DMSO (dimethyl sulfoxide), H89, LY294002 and 8-Br-cAMP were from Sigma (St. Louis, MO). L-NAME, PP2 and Rp-cAMP were from Alexis (San Diego, CA). SNAP (S-nitroso-N-acetyl-DL-penicillamine) and PD98059 were form Caymen Chemical. Compound C was from Merck Millipore. DAF-FM diacetate was from Invitrogen (Carlsbad, CA). The vWF ELISA kit was described previously [45].

### RNA Interference

To silence eNOS, AMPKα1, Akt1/2 and PKA (catalytic subunits α and β), we used a commercial lentiviral system from Sigma to deliver short hairpin RNAs (shRNAs). The target and control scrambled sequences were selected according to an open program (http://jura.wi.mit.edu/bioc/siRNAext/). The shRNA sequence targeting eNOS was 5′-GTGGCCAACGCCGTGAAGATC-3′; for PKA catalytic subunits α and β, 5′-GCTCCCTTCATACCAAAGTTT-3′ and 5′-CACAGCCCACTTGGATCAGTT-3′;for AMPKα1, 5′-GTACGACTAAGCCCAAATCTT-3′; for Akt1 and Akt2, 5′-GGAGGGTTGGCTGCACAAATT-3′ and 5′-CTCCTTGGCAAGGGAACCTTT -3′; and the control scrambled sequence was 5′-CCTAAGGTTAAGTCGCCCTCG-3′.

### Cell Culture

The origin and the culture of human umbilical vein endothelial cells (HUVECs) used in this manuscript have been described in the literature [45], [46]. The cells were cultured at 37°C in a humidified atmosphere of 5% CO_2_. Cells were used from passages 3 to 6. 293T and HL-60 cells were obtained from the ATCC and cultured in Dulbecco’s modified Eagle’s medium (DMEM) containing 10% fetal bovine serum.

### Biaxial Stretch of Cultured Cells

HUVECs were stretched on a device established for the study of static continuous stretch, which has been described in detail [47]. Serum-starved ECs were cultured on rat-tail collagen-coated silicone elastic membrane (Specialty MFG, MI) in a single-well device and were uniformly stretched by vertical indentation, resulting in sustained homogeneous strain of 20–50% (static control as 0%). All experiments were performed under 50% stretch unless otherwise noted.

### ELISA Analysis

Confluent HUVECs were starved in serum-free M199 medium supplemented with 2% BSA for 4 h, and then stimulated by stretch or other factors. The supernatant was harvested and centrifuged at 3600 rpm at 4°C. The concentration of vWF was assessed by the standard sandwich ELISA procedure according to the manufacturer’s instructions.

### Annexin V/PI Staining

Confluent ECs were washed twice with cold PBS, fixed with 4% PFA supplemented with 0.2 mol/L sucrose for 1 hr at 4°C. After washing three times with PBS, ECs were stained with Annexin V/PI kits (Invitrogen V13241) according to the protocol and incubated at room temperature for 15 min and then washed another three times. ECs were also stained with DAPI.

### Virus Preparation and Infection

Preparations of lentiviruses were made in 293T cells. Forty-eight hours after the cells were transfected, the virus-containing supernatant was harvested. Three milliliters of supernatant, mixed with 3 mL fresh M199 medium containing a final concentration of 8 µg/mL polybrene, was used to infect ECs as previously described [37]. The medium was replaced with normal M199 growth medium after 24 h, and HUVECs were harvested for experiments after 72 h infection.

### Western-blot and Immunoprecipitation Analysis

Confluent HUVECs were starved in serum-free M199 medium supplemented with 1% BSA for 16 h before stimulation. The stimulated cells were washed twice in ice-cold PBS and lysed in buffer containing 50 mM HEPES, 150 mM NaCl, 1% Triton X-100, 10% glycerol, 1.5 mM MgCl_2_, 1 mM EGTA, 5 mM EDTA, 0.27 g/mL Na_4_P_2_O_7_, 5 g/mL aprotinin, 1 g/mL prostatin A, 1 g/mL antipan, 10 g/mL leupeptin, 1 mg/mL PMSF, 2 mM beta-glycerol phosphate, 10 mM NaF, and 2 mM Na_3_VO_4_. The lysates were fractionated on 8% SDS-PAGE, followed by standard Western blot analysis.

### Measurement of Nitric Oxide Production

Confluent HUVECs were starved in serum-free M199 medium supplemented with 2% BSA for 3 h, and incubated with 5 µM DAF-FM diacetate in phenol red-free DMEM for 30 min at 37°C. Then the cells were washed gently with PBS and stimulated by stretch. The real-time changes in DAF fluorescence were recorded though a 40×oil-immersion lens and analyzed by laser scanning confocal microscopy (Zeiss, Germany).

### *In vitro* Leukocyte Adhesion Assay

Confluent HUVECs were starved in serum-free M199 medium supplemented with 2% BSA for 4 h, and then stimulated by stretch for 30 min. Then the supernatant was discarded and 2×10^6^ HL-60 cells were added to the surface of the endothelial cells. After 30-min incubation, the unbound cells were washed off 3 times with M199 medium, the bound cells were fixed with 4% paraformaldehyde, and images were captured on a Nikon inverted microscope with a 20× objective lens. The numbers of bound HL-60 cells in each field were counted and analyzed by ANOVA. Representative results of three independent experiments are shown.

### Statistics

Results are expressed as mean ± S.D. on the basis of triplicate experiments. Statistical analysis was done using ANOVA with Bonferroni’s correction. A value of *P*<0.05 was considered statistically significant.

## Supporting Information

Figure S1 **Effects of stretch on the survival and death of HUVECs.** (A) Immunofluorescence staining of Annexin V/PI and DAPI in HUVECs stretched for 1 hr–8 hrs. HUVECs starved in empty M199 medium served as positive controls. (B) Quantitative analysis of trypanblue staining of HUVECs stretched for 1 hr–8 hrs. Results are representative of 3 individual experiments and expressed as mean ± SD (n = 4). *P<0.05; N.S., not significant.
(TIF)
