# Supplementary figures and images for: Acute Mechanical Stretch Promotes eNOS Activation in Venous Endothelial Cells Mainly via PKA and Akt Pathways
Source: PLoS One. 2013 Aug 14;8(8):e71359. doi: 10.1371/journal.pone.0071359 (PMC3743752; doi:10.1371/journal.pone.0071359)

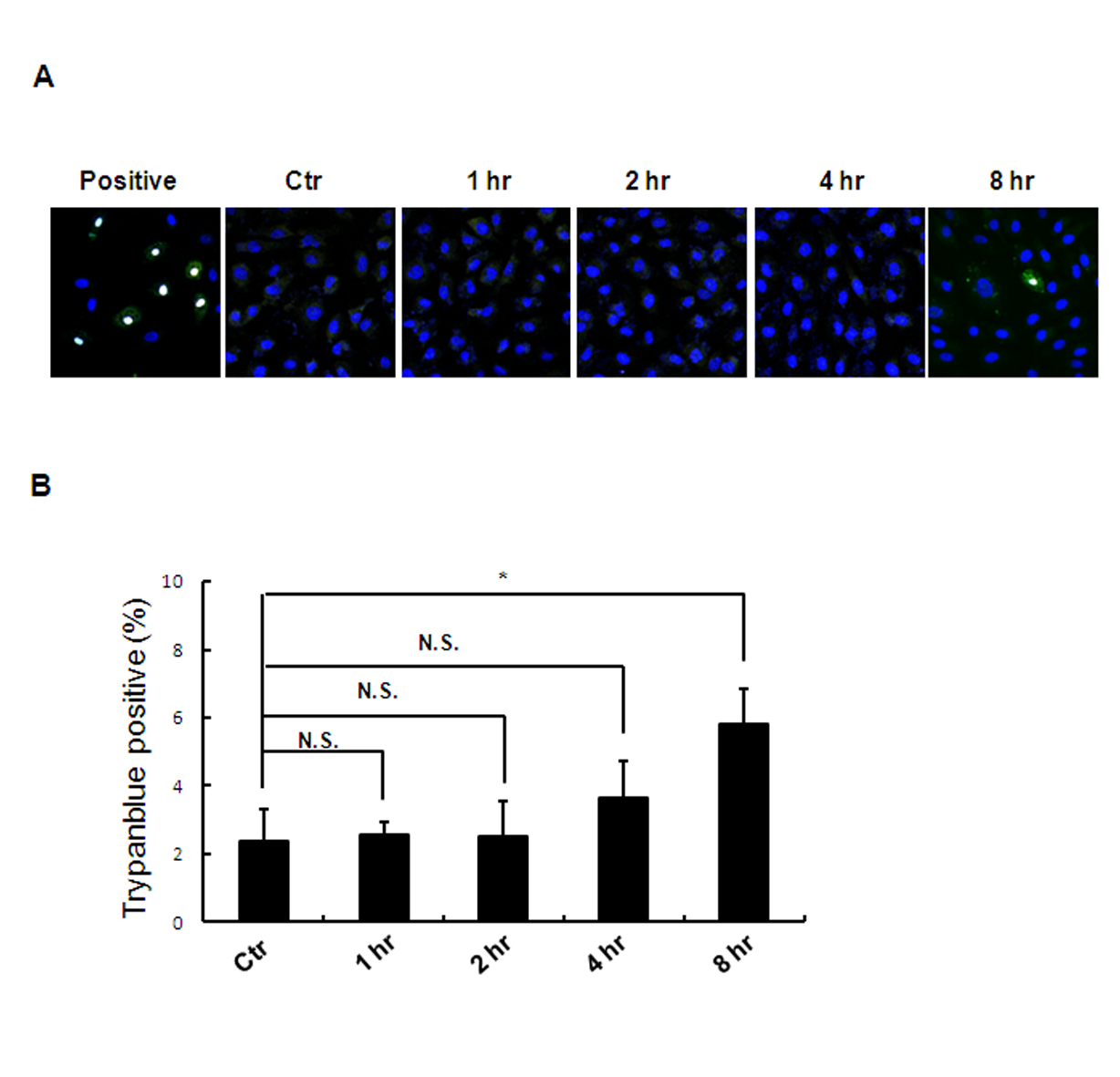

Supplement: Figure S1 — Effects of stretch on the survival and death of HUVECs. (A) Immunofluorescence staining of Annexin V/PI and DAPI in HUVECs stretched for 1 hr–8 hrs. HUVECs starved in empty M199 medium served as positive controls. (B) Quantitative analysis of trypanblue staining of HUVECs stretched for 1 hr–8 hrs. Results are representative of 3 individual experiments and expressed as mean ± SD (n = 4). *P<0.05; N.S., not significant. (TIF) [file pone.0071359.s001.tif]
